# Validation of Aspartylglucosaminidase Activity Assay for Human Serum Samples: Establishment of a Biomarker for Diagnostics and Clinical Studies

**DOI:** 10.3390/ijms24065722

**Published:** 2023-03-16

**Authors:** Antje Banning, Minna Laine, Ritva Tikkanen

**Affiliations:** 1 Institute of Biochemistry, Medical Faculty, University of Giessen, Friedrichstrasse 24, DE-35390 Giessen, Germany; 2 Department of Child Neurology, Helsinki University Hospital and Helsinki University, P.O. Box 900, FI-01400 Vantaa, Finland

**Keywords:** aspartylglucosaminuria, lysosomal storage disorders, biomarkers, protein glycosylation, enzymes

## Abstract

Novel treatment strategies are emerging for rare, genetic diseases, resulting in clinical trials that require adequate biomarkers for the assessment of the treatment effect. For enzyme defects, biomarkers that can be assessed from patient serum, such as enzyme activity, are highly useful, but the activity assays need to be properly validated to ensure a precise, quantitative measurement. Aspartylglucosaminuria (AGU) is a lysosomal storage disorder caused by the deficiency of the lysosomal hydrolase aspartylglucosaminidase (AGA). We have here established and validated a fluorometric AGA activity assay for human serum samples from healthy donors and AGU patients. We show that the validated AGA activity assay is suitable for the assessment of AGA activity in the serum of healthy donors and AGU patients, and it can be used for diagnostics of AGU and, potentially, for following a treatment effect.

## 1. Introduction

Aspartylglucosaminuria (AGU, OMIM 208400) is a neurodevelopmental disease caused by the deficiency of a lysosomal hydrolase, aspartylglucosaminidase (AGA, EC 3.5.1.26). AGU patients show a progressive but relatively slow cognitive decline, which may prevent an early diagnosis. Beyond the age of 20 to 25, AGU patients are usually severely cognitively impaired and may also exhibit physical deterioration and profound handicap. The life expectancy of the patients is higher than 50 years of age, and the oldest AGU patients currently alive are over 60 (reviewed in [1,2]).

AGU is enriched in the Finnish population, and due to a founder effect, almost all Finnish patients carry a specific double missense variant, termed AGU_Fin-major_, in at least one allele [3,4]. This variant consists of a neutral amino acid exchange, Arg161Gln, combined with a pathogenic Cys163Ser exchange that prevents the formation of a disulfide bond and results in a folding defect [5]. Currently, there are no approved therapies for AGU, although several potential treatments have been assessed in preclinical studies [6,7,8,9].

In Finland, AGU is usually diagnosed after analysis of urine oligosaccharides, especially N-acetylglucosamine-asparagine (GlcNAc-Asn), and the diagnosis is verified by means of gene sequencing [10,11]. The patients outside of Finland, who often exhibit their own, family-specific pathogenic variants, are identified either by gene/exome sequencing, or urine analysis. Measurement of AGA activity in white blood cells has also been used for diagnostic means or for the verification of previous findings [12,13,14,15], but this method is rarely available in routine diagnostic laboratories. Nevertheless, findings of a genetic analysis or urine glycoasparagine measurement should always be verified by a second method, especially if a novel, potentially pathogenic variant of the *AGA* gene is identified [10]. Furthermore, glycoasparagines can also be present in the urine of patients with *NGLY1* deficiency, which is a related neurodevelopmental disorder [11,16,17,18].

In the era of emerging treatment strategies, such as pharmacological chaperones, gene therapy and enzyme replacement therapy for rare genetic disorders, including AGU, there is a high need for validated biomarkers that can be used to measure the effect of the potential treatments. Optimally, such biomarkers can be obtained in a minimally invasive fashion. For AGU, accurate measurement of AGA activity in serum or plasma samples would be highly desirable, in addition to analysis of urine GlcNAc-Asn.

The purpose of this study was to establish and validate an AGA enzyme activity assay for human serum samples. We here provide a validated method for the measurement of AGA activity from human serum samples, for use as a biomarker for diagnostic purposes and, potentially, for the assessment of treatment efficiency.

## 2. Results

### 2.1. Validation of the AGA Enzyme Activity Assay

The development of an optimized method for the determination of AGA activity in AGU patient sera was based on previously published methods [6,7,8,11,15]. The measurement of AGA enzyme activity in human fibroblasts and leucocytes using a fluorometric substrate Asp-AMC (L-Aspartic acid β-(7-amido-4-methylcoumarin)) was originally proposed by Mononen et al. and Voznyi et al. [13,14,15,19]. The suitability of Asp-AMC as an AGA substrate for the measurement of AGA activity in clinical samples (e.g., serum, plasma and leucocytes) has also been described by Mononen et al. [13]. In addition, we have previously used this activity assay for biological samples, such as cell lysates [6,7,8,20]. However, the method for a quantitative measurement of AGA activity has not been validated for clinical use for human serum.

Due to these reasons, it was necessary to further optimize and validate the AGA activity assay for human serum samples. According to the European Medicines Agency (EMA) guidelines on bioanalytical method validation, the imprecision and inaccuracy of an analytical method should be lower than ±15%, with the only exception of the lower limit of quantification (LLOQ), for which a deviation of ±20% is allowed [21]. The validation of the AGA activity measurement from human serum samples (healthy donors and AGU patients) was undertaken by testing various parameters, as described in the following sections.

#### 2.1.1. Linearity and Lower Limit of Quantification

**Figure 1:**
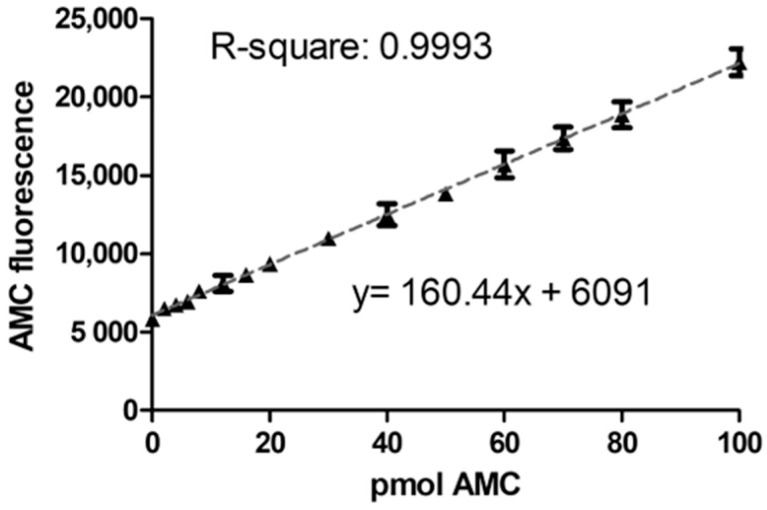
Linearity of the AMC standard curve. AMC-standards were prepared as outlined in Section 4.4. Raw AMC fluorescence (in artificial units) was plotted against the corresponding AMC amount in pmol. The slope, y-intercept, and R-square were calculated by linear regression analysis. Symbols show the mean ± SD of five independent analyses.

For the calibration standards, 7-Amino-4-methylcoumarin (AMC), the fluorescent product that arises from the substrate Asp-AMC in the AGA enzymatic reaction, was used. According to the guideline [21], the calibration standards should be prepared in the same matrix as the matrix of the samples of interest. Therefore, 14 calibration samples with different AMC amounts were prepared in a commercially available, artificial serum matrix. The total amount of AMC in the calibration standards ranged from 0 to 100 pmol. Five separate runs were performed with the calibration standards, and each calibration curve was fitted by linear regression. The measured concentrations of the calibration standards were back-calculated to the expected nominal value. The five individual calibration curves (Supplementary Figure S1) were averaged (Figure 1), with the average equation represented as: y = 160.44x + 6091, R-square = 0.99. For each of the five calibration curves, at least 11 standards (i.e., more than 75%) fulfilled the criteria of back-calculation within the ±15% of the nominal value range. The average LLOQ was calculated to be 4.8 pmol AMC (corresponding to 0.18 mU/L AGA activity), but the lowest AMC-containing standard with 2 pmol AMC (0.09 mU/L) could also be detected. However, the back-calculation of this standard was not within the required ±20% range, indicating that measuring samples with enzyme activities as low as 0.09 mU/L is still possible, but less precise.

#### 2.1.2. Accuracy

**Figure 2:**
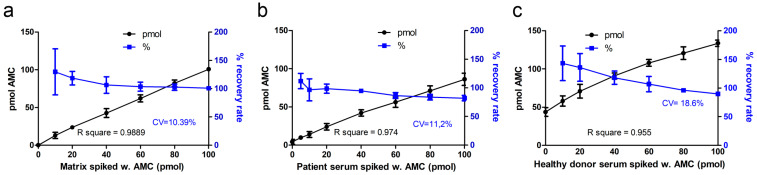
Accuracy of AMC detection. Inter-assay accuracy was determined by spiking of samples with defined amounts of AMC. (**a**) artificial serum matrix; (**b**) AGU patient serum, or (**c**) serum of healthy donors. The samples were supplemented with 10–100 pmol of AMC and incubated for 24 h to mimic the AGA activity assay protocol. Nominal and measured AMC values were plotted against each other (black symbols, left axis). R-square was calculated by linear regression analysis, and recovery rate (blue symbols, right axis) was computed from the determined value and expressed as % from the true value. The values represent the mean ± SD of three independent experiments.

**Figure 3:**
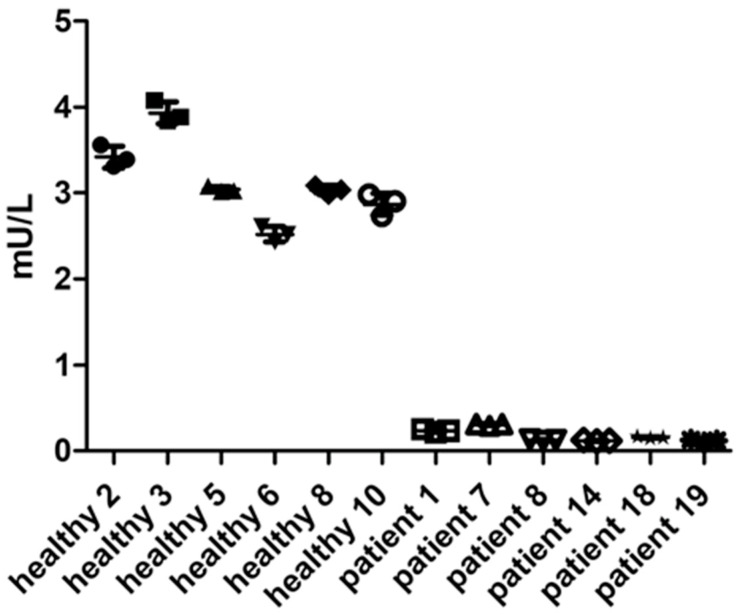
Within-run precision. AGA activity in three technical replicates with 10 µL serum from 6 healthy donors or 20 µL of serum from 6 AGU patients was measured following the validated AGA activity assay protocol. Data show one representative run out of at least 3 runs.

Inter-assay accuracy was determined by spiking of samples (i.e., artificial serum, patient serum, healthy donor serum) with known amounts of AMC. Artificial serum matrix was spiked with 10 to 100 pmol AMC, and the resulting fluorescence was measured (Figure 2a). Recovery rate for most of the AMC standards spiked in the artificial serum was on average 106%, with coefficient of variation (CV) of 10.39%. Only for the lowest spiked amount of AMC (10 pmol), the recovery rate (130%) was not within the allowed 15% range. The R-square for all AMC amounts was 0.9889. The recovery rate for AMC spiked in patient sera was on average 93%, with CV 11.2% (Figure 2b). However, AMC spiked in healthy donor serum samples (Figure 2c) showed a higher variation (CV 18.6%) of the recovery rate (115%), which was mainly due to the low accuracy of the lowest amount of spiked AMC (10 pmol). Since the AGA activity in healthy donors is already in the upper range of the calibration curve (see Figure 3), the detection of spiked AMC in such samples would require an extrapolation of the calibration curve. 

#### 2.1.3. Precision

Next, the precision of the AGA activity assay was determined using clinical samples of healthy donors and AGU patients, and the AGA activity was measured using the substrate Asp-AMC. For the measurements, 12.5 µM substrate Asp-AMC was incubated for 24 h with the serum samples (Figure 3). Time-scale analysis showed that the activity measurement was linear within the time-scale of 1 to 24 h (Supplementary Figure S2).

Precision describes the degree of scatter of repeated measurements and is expressed as CV. Both accuracy and precision should optimally be within ±15%, except for the LLOQ, for which ±20% is considered acceptable [21]. Within-run precision was determined using serum samples from six healthy donors, representing the “high” quality control (QC) samples, and six AGU patients, representing the “low” QC samples, each measured in triplicates (Figure 3). Average CV was 4.96%, and all subjects were within the required ±15% precision range, demonstrating that the AGA activity assay is suitable for measuring the AGA activity even in patient samples (Supplementary Table S1).

**Figure 4:**
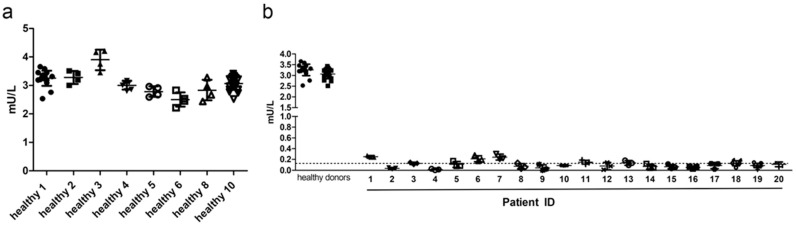
Between-run precision. (**a**) Between-run precision of the AGA activity assay was assessed with serum samples of 8 healthy donors. Each symbol represents one independent measurement out of at least 4 experiments. (**b**) Between-run precision of the AGA activity assay in AGU patient sera. Samples from 20 AGU patients were assessed by three independent experiments. The data for two healthy donors from panel (**a**) are shown for comparison.

Between-run precision of at least three individual runs was assessed with serum samples of eight healthy donors (Figure 4a) and 20 AGU-patients (Figure 4b). Average CV was 8.25% for healthy donors, and all healthy donors passed the quality criteria. Most patient samples showed CV higher than 20%, which is caused by the very low AGA activity, often being even below the determined LLOQ (dotted line in Figure 4b). Supplementary Table S1 shows a summary of the individual coefficients of variation.

All 20 patient samples displayed severely reduced AGA activities that were on average 0.11 mU/L (range 0.0123–0.251 mU/L). Healthy donors typically had AGA activities on average 3.252 mU/L (range 2.503–3.897 mU/L). With our assay, the diagnosis of an AGU patient is thus unambiguous, and a misdiagnosis is very unlikely.

#### 2.1.4. Dilution Integrity

**Figure 5:**
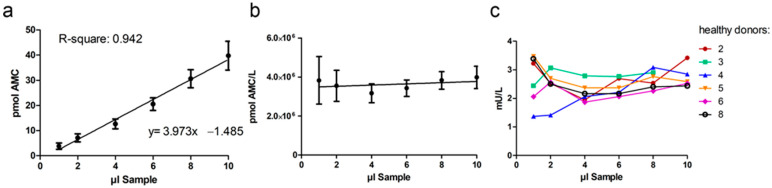
Dilution integrity. AGA activity was measured with different amounts of serum (1–10 µL) from healthy donors. All samples were diluted with NaCl or artificial serum to 20 µL total volume for the assay. AGA activity in the serum samples of six healthy donors was measured according to the validated protocol. (**a**) The mean values (pmol AMC) of the six healthy donors were used for linear regression analysis. (**b**) The mean AMC values back-calculated as pmol AMC per liter over the sample volume range; (**c**) Individual AGA activities (mU/L) in the serum samples of the six healthy donors.

Dilution of the samples should not affect the accuracy and precision of a method. Here, dilution integrity was demonstrated by measuring the AGA activity from 1 to 10 µL of serum from six healthy donors. The volume of the samples was adjusted to 20 µL with isotonic NaCl or the artificial serum matrix. Dilution of patient samples was not carried out, since the activity with 20 µL of patient serum is already at the detection limit (see Figure 4b). Dilution of the sera from healthy donors showed that the assay was precise even for serum volumes as low as 4 µL (Figure 5a–c). With volumes less than 4 µL serum, the precision decreased (Figure 5b).

## 3. Discussion

The main purpose of this study was to establish and validate a method for a quantitative measurement of AGA activity in human serum samples. The fluorometric method that was used as a starting point was originally developed and then further refined by Mononen et al. and Voznyi et al. [13,14,15]. Before that, a colorimetric assay was used that is less sensitive and more error-prone than the fluorometric method [22,23]. Voznyi et al. have used the fluorometric method for the assessment of AGA activity also from amniotic fluid, amniocytes, chorionic villi biopsies and cultured trophoblasts for prenatal diagnostics [15]. However, the methods have so far not been validated for a precise measurement of AGA activity in human samples, and they mainly allow the distinction of AGU patients from healthy individuals or carriers. For example, Mononen et al. showed that the identification of carriers by a measurement of AGA activity in serum samples was not possible with the previous methods [13]. Furthermore, as AGA activity in the AGU patients is very low, the activity measurement needs to be carefully validated for the low activity range so that effect of potential treatments such as gene therapy can be accurately assessed in clinical trials. Therefore, we here validated the AGA activity measurement for human serum samples, using both healthy donors and AGU patients.

In the previous published protocols for cell lysates and serum, very high Asp-AMC concentrations (0.5 to 1.5 mM) have been used [13,14,15]. However, these concentrations produced a high background fluorescence when measured with our plate reader and were thus not suitable for the measurement in the low activity range, i.e., in patient samples. Hence, already in our previous cell culture studies with AGU patient fibroblasts and loss- and gain-of-function cell lines, we reduced the Asp-AMC concentration to 25 µM [6,8,13,14,15,24]. However, even then, the method had limitations when comparing the AGA activity of samples with very low enzyme activity, so we here further reduced the Asp-AMC concentration to 12.5 µM. Further optimization of the method for human serum samples that have AGA activities close to the LLOQ, as systematically defined here, was performed.

Our validated activity assay differs from the previously published assays in several ways. The buffer system we have used is similar to the one used by Voznyi et al. (Mc Ilvain’s phosphate-citrate buffer) [15], but we used a commercially available, artificial serum matrix for the blanks, standards, and for sample dilution. As compared to the assay published by Mononen et al. [14], we have used a longer incubation time (24 h vs. 1 h) to reduce the variation in the low activity range.

For the validation, we used AMC standards diluted in artificial serum, for which the LLOQ was determined to be 4.8 pmol AMC (corresponding to 0.18 mU/L AGA activity). However, also the lowest AMC-containing standard with 2 pmol AMC (0.09 mU/L) was detectable, albeit with a lower accuracy. We noticed that it was necessary to use an artificial serum instead of a buffer for the dilution, as this resulted in more comparable background counts when measuring serum samples.

In the patient samples, the AGA activity was within the range of 0.0123–0.251 mU/L (average 0.11 mU/L). Although some patients fall below the LLOQ, they can be clearly distinguished from healthy donors, who show an average AGA activity of 3.252 mU/L. Therefore, a diagnosis of AGU can unambiguously be made from serum samples using our validated assay. Mononen et al. obtained mean AGA enzyme activities of 20.2 ± 5.0 mU/L for healthy donor sera [13], which is considerably higher than the average 3.252 mU/L in our study. This difference is most likely due to the very different substrate concentrations used in the assays. Mononen et al. used 500 µM Asp-AMC, whereas our assay uses 12.5 µM substrate (i.e., 40 times less). However, the difference in the calculated AGA activities is not due to the prolonged incubation time (in our case 1440 min), as our assay is linear over the time course of 1 h to 24 h, consistent with the data of Voznyi et al. [15]. In addition, the AGA activities in the sera of healthy donors was about 29 times that of the AGU patients both in the present study and in Mononen et al. [13], demonstrating that even though the calculated AGA activities vary due to the assay conditions, the relative difference between the healthy donors and AGU patients stays the same.

All patients in our study were homozygous for the major Finnish variant. However, most AGU patients with variants other than AGU_Fin-major_ are also expected to fall within the same activity range and can thus be identified with our assay. So far, only few AGU patients have been identified who show an intermediate AGA activity that is higher than that in the Finnish patients, but lower than that in the carriers [7]. However, our activity assay allows the identification of these patients, too, even if they fall in the range above LLOQ. Unfortunately, medium QC samples, such as samples from non-affected carriers (e.g., parents of AGU-patients), were not available for our study, but they were mimicked by dilution of high QC samples from healthy donors. Our data show that expected carrier activities can also be precisely measured, and dilution of the samples did not affect the precision of the method, since an amount as little as 4 µL of healthy donor serum was sufficient for an accurate AGA activity measurement. However, due to the low activity in AGU patients, 20 µL of patient serum is required.

The within-run precision (triplicate measurements) for the AGA activity was accurate (below the 15% variation range), even for most of the AGU patient samples. However, for samples at or below LLOQ, ±20% variation is actually considered acceptable. The between-run variation of at least three individual measurements for healthy donor samples was very good (CV 8.25%). Most of our 20 AGU patients, however, were above the 20% variation limit between the runs. Thus, it can be concluded that the precision of the method in AGU patient samples is acceptable for intra-assay replicates, but not always for inter-assay analysis. Therefore, patient samples that need to be directly compared with each other, for example when treatment effects are longitudinally measured, should preferably be analyzed in the same run. Future data from clinical studies on AGU, such as our study registered under EudraCT number 2017-000645-48 [25], will reveal if the activity assay described here will be capable of measuring the changes in AGA activity as a result of an interventional treatment.

## 4. Materials and Methods

### 4.1. Sample Collection

Blood was collected from healthy donors via venipuncture. After 15 min at room temperature, samples were centrifuged at 4 °C for 10 min at 390× *g*. Serum samples from 20 AGU patients were obtained within the framework of an interventional clinical study (EudraCT number 2017-000645-48) [25], before any intervention was started. All serum samples were divided in aliquots and stored at −80 °C until analysis. Prior to use, the aliquots were thawed on ice and mixed well to dissolve any precipitated material. 

### 4.2. Reagents

The AGA substrate Asp-AMC (Cat. sc-211699) was obtained from Santa Cruz Biotechnology (Heidelberg, Germany). The substrate was dissolved in 1 M HCl as 1 mM stock solution. Out of this stock solution, the 25 µM substrate solution was prepared in McIlvain’s phosphate-citrate buffer, pH 6.5. McIlvain’s phosphate-citrate buffers, pH 4.5 and 6.5 were prepared by mixing 5.46 mL or 2.9 mL of 1 M citric acid with 18.16 mL or 28.4 mL of 0.5 M Na_2_HPO_4_, respectively. Water was added to 100 mL, and the pH was adjusted, if necessary.

Artificial serum/plasma II (Cat. ASP-002) was obtained from Biopanda Diagnostics (Belfast, UK). Isotonic NaCl solution (0.9%) was from B. Braun (Melsungen, Germany) and AMC (7-Amino-4-methylcoumarin, Cat. A9891) was from Sigma-Aldrich (Taufkirchen, Germany). AMC was dissolved in DMSO at 10 mg/mL, and the AMC standards were prepared as 5 µM dilution in the artificial serum. AMC and Asp-AMC stocks and working solutions were stored at −20 °C.

### 4.3. Validation of the Enzyme Activity Measurement

Experimental validation of the AGA activity measurement from human serum samples was performed using EMA guidelines [21]. As a starting point, our published AGA activity protocol for cell culture lysates was used [6,7], which was here adapted and validated for human serum samples. The following parameters were validated:

#### 4.3.1. Linearity and Lower Limit of Quantification (LLOQ)

Calibration samples were prepared in the artificial serum matrix and analyzed in five separate runs. Each calibration sample contained a defined amount of AMC and Asp-AMC. The sum of AMC and Asp-AMC was kept constant at 12.5 µM (Table 1). All samples were incubated for 24 h at 37 °C after what 200 µL of McIlvain’s phosphate-citrate buffer, pH 4.5, was added. All tubes were vortexed, and 200 µL out of each tube were pipetted into a black 96-well plate. The plates were measured (excitation: 355 nm, emission: 460 nm) with a Tecan Infinite M200 (Tecan, Männedorf, Switzerland) plate reader (gain 120, 4 × 4 reads per well). Each calibration curve was fitted by linear regression. Slope, Y-intercept, and R-square were calculated using Microsoft Excel 2016 (Microsoft Corp., Munich, Germany). Concentrations of the calibration standards were back-calculated and should be within ±15% of the nominal value, except for LLOQ, which should be within ±20%. The five individual calibration curves were averaged, and the average LLOQ was calculated.

#### 4.3.2. Accuracy and Precision

Accuracy describes the closeness of the obtained value to the nominal value, reported as %, and is determined by spiking of samples with known amounts of the measured analyte (i.e., AMC). Accuracy was determined in artificial serum, in patient serum, and in healthy donor serum. Furthermore, 20 µL of artificial serum, or patient serum, or 10 µL of healthy donor serum were spiked with 10–100 pmol AMC. Then, 20 µL of Asp-AMC was added, and the samples were incubated for 24 h at 37 °C. Thereafter, the reaction was stopped by adding 200 µL of McIlvain’s phosphate-citrate buffer pH 4.5. Fluorescence measurements were performed as described for the calibration standards. Accuracy was calculated as determined value expressed as percentage of the true value. Between-run accuracy was evaluated by analyzing the variation coefficient of three independent experiments.

Precision is an indication of the degree of scatter of repeated measurements and is defined as (standard deviation /mean) × 100%. Within-run precision was determined for six healthy donors and six AGU patients, each measured in triplicates. Between-run precision of at least three different runs was assessed for eight healthy donors and 20 AGU-patients. Both accuracy and precision should be within ±15%, except for the LLOQ (±20%).

#### 4.3.3. Dilution Integrity, Carry-Over and Stability

Dilution integrity was demonstrated with 1–10 µL of serum from healthy donors. The volume difference was balanced with isotonic NaCl or artificial serum matrix. R-square and coefficient of variation were calculated with GraphPad Prism 5 (San Diego, CA, USA).

Carry-over of the analyte does not occur, because all samples are pipetted and measured independently of each other. AMC and Asp-AMC aliquots were stored at −20 °C and used several times, without loss of potency due to degradation. Serum samples were stored at −80 °C. Freezing and thawing for at least three times did not result in any obvious loss of AGA enzyme activity (See Figure 4 and Supplementary Figure S1).

### 4.4. Validated Method for the Measurement of AGA Activity in Human Serum

**Figure 6:**
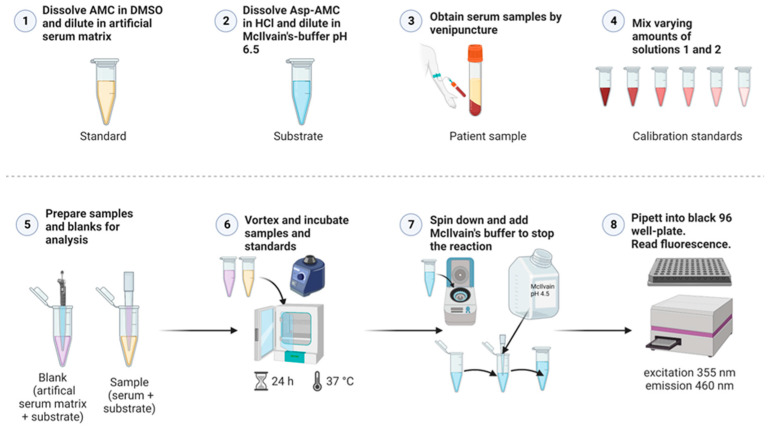
Schematic presentation of the steps of the AGA activity assay. See Section 4.4 for details.

Figure 6 shows a general scheme for the AGA activity assay. For all samples, three 0.5 mL tubes were prepared with 20 µL of 25 µM Asp-AMC substrate solution. In these tubes, 20 µL patient serum was added, whereas for healthy donors, 10 µL serum and 10 µL isotonic NaCl were added. Triplicate blank samples were prepared with 20 µL artificial serum and 20 µL Asp-AMC substrate solution. Note that the blanks are identical to the calibration sample S1 of the standard curve (Table 1). For the AMC standard curve, 16 tubes were prepared, containing increasing amounts of AMC in artificial serum, ranging from 0 to 100 pmol AMC. Because of the background fluorescence of Asp-AMC, defined amounts of Asp-AMC substrate solution were added, so that the total amount of AMC and Asp-AMC was identical in all standards. Artificial serum was added to 40 µL (see Table 1 for detailed pipetting scheme). All tubes were tightly closed, vortexed and incubated at 37 °C for 24 h. After 24 h, all tubes were centrifuged for 10 s to collect any condensed fluid. Reactions were stopped by addition of 200 µL of McIlvain’s phosphate-citrate buffer, pH 4.5. All tubes were vortexed, 200 µL from each tube were transferred into a black 96-well plate. The plates were measured immediately (excitation: 355 nm, emission: 460 nm) with a Tecan Infinite M200 plate reader (gain 120, 4 × 4 reads per well). For the calculation of AGA enzyme activity, the mean blank fluorescence was subtracted from all samples. According to the slope of the standard curve, the AGA activity values are expressed as mU/L (1 mU = 1 nmol/min, 24 h corresponding to 1440 min). The enzyme activity (mU/L) is calculated from the amount of the liberated product (AMC), taking into account the sample volume and the incubation time:

Enzyme activity in mU/L = nmol AMC/(1440 min × sample volume in L)

## Figures and Tables

**Table 1:** Pipetting scheme for calibration standards.

Standard	AMC pMol	AMC µM	µL AMC (5 µM) in Artificial Serum	Asp-AMC µM	µL Asp-AMC (25 µM) in McIlvain’s pH6.5	Asp-AMC + AMC µM	µL Artificial Serum
S0	0	0	0.00	0	0.00	0	20 *
S1	0	0	0.00	12.5	20.00	12.5	20.00
S2	2	0.05	0.4	12.45	19.92	12.5	19.68
S3	4	0.1	0.80	12.4	19.84	12.5	19.36
S4	6	0.15	1.20	12.35	19.76	12.5	19.04
S5	8	0.2	1.60	12.3	19.68	12.5	18.72
S6	12	0.3	2.40	12.2	19.52	12.5	18.08
S7	16	0.4	3.20	12.1	19.36	12.5	17.44
S8	20	0.5	4.00	12	19.20	12.5	16.80
S9	30	0.75	6.00	11.75	18.80	12.5	15.20
S10	40	1	8.00	11.5	18.40	12.5	13.60
S11	50	1.25	10.00	11.25	18.00	12.5	12.00
S12	60	1.5	12.00	11	17.60	12.5	10.40
S13	70	1.75	14.00	10.75	17.20	12.5	8.80
S14	80	2	16.00	10.5	16.80	12.5	7.20
S15	100	2.5	20.00	10	16.00	12.5	4.00

* +20 µL McIlvain’s pH 6.5.

## Data Availability

Primary data are available from the corresponding author upon a justified request.

## Supplementary Materials

The following supporting information can be downloaded at: https://www.mdpi.com/article/10.3390/ijms24065722/s1.
