# Supplementary material for: Validation of Aspartylglucosaminidase Activity Assay for Human Serum Samples: Establishment of a Biomarker for Diagnostics and Clinical Studies
Source: Int J Mol Sci. 2023 Mar 16;24(6):5722. doi: 10.3390/ijms24065722 (PMC10059667; doi:10.3390/ijms24065722)
Supplement: Supplementary file 1 [file ijms-24-05722-s001.zip › ijms-2253035-supplementary.pdf]

Supplementary Figure S1

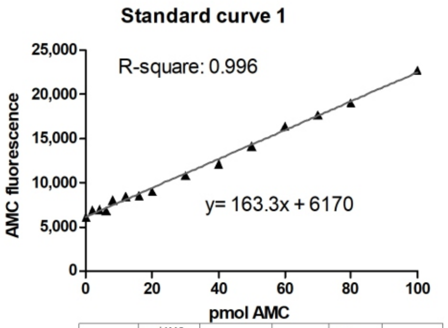

	pmol AMC in serum matrix	Fluorescence	pmol AMC	% of expected value	% deviation
S1	0	6,138	-0.23		
S2	2	6,891	4.39	219.35	119.35
S3	4	6,943	4.71	117.64	17.64
S4	6	6,814	3.92	65.25	-34.75
S5	8	8,026	11.34	141.77	41.77
S6	12	8,466	14.04	116.98	16.98
S7	16	8,544	14.52	90.72	-9.28
S8	20	9,042	17.57	87.83	-12.17
S9	30	10,825	28.49	94.97	-5.03
S10	40	12,153	36.63	91.57	-8.43
S11	50	14,125	48.71	97.42	-2.58
S12	60	16,427	62.81	104.69	4.69
S13	70	17,686	70.53	100.76	0.76
S14	80	19,027	78.75	98.43	-1.57
S15	100	22,770	101.68	101.68	1.68

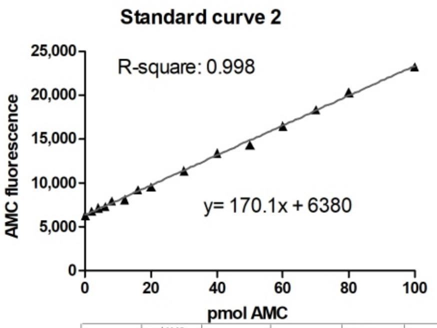

	pmol AMC in serum matrix	Fluorescence	pmol AMC	% of expected value	% deviation
S1	0	6,347	-0.22		
S2	2	6,805	2.47	123.53	23.53
S3	4	7,199	4.79	119.71	19.71
S4	6	7,325	5.53	92.16	-7.84
S5	8	7,984	9.41	117.57	17.57
S6	12	8,134	10.29	85.74	-14.26
S7	16	9,255	16.88	105.51	5.51
S8	20	9,603	18.93	94.65	-5.35
S9	30	11,412	29.57	98.57	-1.43
S10	40	13,424	41.41	103.51	3.51
S11	50	14,367	46.95	93.91	-6.09
S12	60	16,558	59.84	99.74	-0.26
S13	70	18,411	70.74	101.06	1.06
S14	80	20,328	82.02	102.52	2.52
S15	100	23,254	99.23	99.23	-0.77

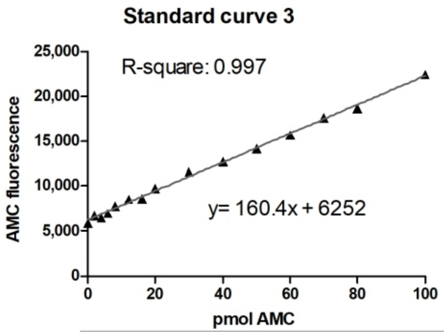

	pmol AMC in serum matrix	Fluorescence	pmol AMC	% of expected value	% deviation
S1	0	5,879	-2.74		
S2	2	6,744	2.70	135.22	35.22
S3	4	6,518	1.28	32.08	-67.92
S4	6	6,995	4.28	71.38	-28.62
S5	8	7,743	8.99	112.34	12.34
S6	12	8,532	13.95	116.25	16.25
S7	16	8,570	14.19	88.68	-11.32
S8	20	9,753	21.63	108.14	8.14
S9	30	11,588	33.17	110.57	10.57
S10	40	12,726	40.33	100.82	0.82
S11	50	14,147	49.26	98.53	-1.47
S12	60	15,702	59.04	98.41	-1.59
S13	70	17,638	71.22	101.74	1.74
S14	80	18,697	77.88	97.35	-2.65
S15	100	22,423	101.31	101.31	1.31

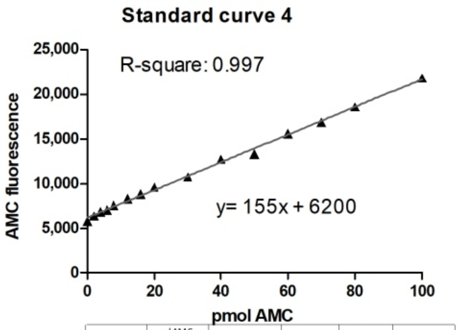

	pmol AMC in serum matrix	Fluorescence	pmol AMC	% of expected value	% deviation
S1	0	5,803	-3.01		
S2	2	6,343	0.50	25.00	-75.00
S3	4	6,875	3.95	98.86	-1.14
S4	6	7,064	5.18	86.36	-13.64
S5	8	7,582	8.55	106.82	6.82
S6	12	8,305	13.24	110.34	10.34
S7	16	8,874	16.94	105.84	5.84
S8	20	9,599	21.64	108.21	8.21
S9	30	10,784	29.34	97.79	-2.21
S10	40	12,744	42.06	105.16	5.16
S11	50	13,292	45.62	91.25	-8.75
S12	60	15,643	60.89	101.48	1.48
S13	70	16,852	68.74	98.20	-1.80
S14	80	18,606	80.13	100.16	0.16
S15	100	21,847	101.18	101.18	1.18

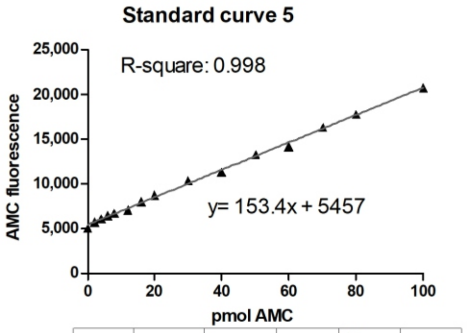

	pmol AMC in serum matrix	Fluorescence	pmol AMC	% of expected value	% deviation
S1	0	5,081	-2.88		
S2	2	5,759	1.58	78.95	-21.05
S3	4	6,121	3.96	99.01	-0.99
S4	6	6,501	6.46	107.68	7.68
S5	8	6,713	7.86	98.19	-1.81
S6	12	7,111	10.47	87.28	-12.72
S7	16	8,025	16.49	103.04	3.04
S8	20	8,806	21.63	108.13	8.13
S9	30	10,366	31.89	106.29	6.29
S10	40	11,327	38.21	95.53	-4.47
S11	50	13,308	51.24	102.49	2.49
S12	60	14,206	57.15	95.25	-4.75
S13	70	16,366	71.36	101.95	1.95
S14	80	17,760	80.53	100.67	0.67
S15	100	20,771	100.34	100.34	0.34

Supplementary Figure S1: Linearity and lower limit of quantification (LLOQ). AMC-standards were pipetted as outlined in Table 1. Raw AMC fluorescence (in artificial units) was related to the corresponding AMC amount in pmol. The slope, y-intercept and, R-square were calculated by linear regression analysis. The data for the independent experiments used for Figure 1 are shown. Concentrations of the calibration standards were back-calculated. Green numbers indicate back-calculation of the standards within the requested limits (± 15% of the nominal value, for LLOQ ± 20%,), yellow colors refer to back calculations close, but outside the requested range, and red colors indicate values where back-calculation was inaccurate. LLOQ of each standard curve was determined to range between 2 and 6 pmol (= uppermost green AMC standard) and was averaged to obtain the mean LLOQ (i.e. 4.8 pmol AMC).

Supplementary Figure S2

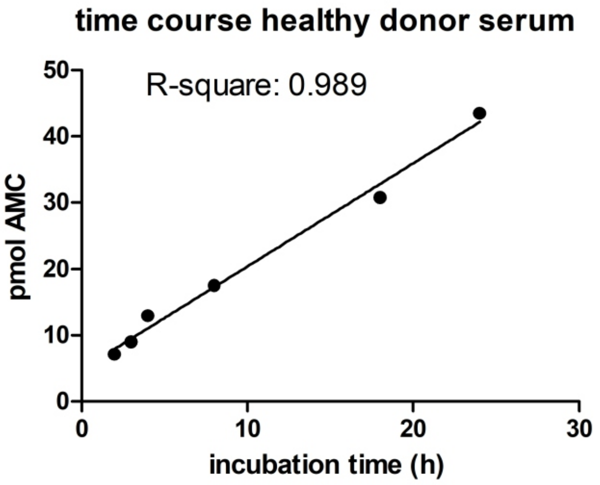

Supplementary Figure S2: Linearity of the activity assay from 1 h to 24 h. AGA activity was measured using 10 µL of serum from a healthy donor. The graph shows the amount of liberated AMC (pmol) as a function of time.

Supplementary Table S1: Coefficients of variation (CV) of the individual measurements of within-run and between-run precision.

Within-run precision (Data from Figure 3)		Between-run precision healthy donors (Data from Figure 4A)		Between-run precision Patients (Data from Figure 4B)	
**Sample ID**	**CV (%)**	**Sample ID**	**CV (%)**	**Sample ID**	**CV (%)**
Healthy donor 2	3.73	Healthy donor 1	8.21	Patient 1	9.81
Healthy donor 3	3.22	Healthy donor 2	6.98	Patient 2	48.94
Healthy donor 5	1.06	Healthy donor 3	9.25	Patient 3	17.63
Healthy donor 6	3.57	Healthy donor 4	4.93	Patient 4	105.82
Healthy donor 8	1.81	Healthy donor 5	6.54	Patient 5	52.14
Healthy donor 10	4.45	Healthy donor 6	10.09	Patient 6	26.19
Patient 1	6.83	Healthy donor 8	12.76	Patient 7	22.31
Patient 7	3.68	Healthy donor 10	7.23	Patient 8	65.24
Patient 8	5.22			Patient 9	128.58
Patient 14	5.98			Patient 10	19.74
Patient 18	5.16			Patient 11	23.66
Patient 19	14.78			Patient 12	86.78
				Patient 13	32.81
				Patient 14	66.66
				Patient 15	62.11
				Patient 16	43.14
				Patient 17	65.04
				Patient 18	37.23
				Patient 19	47.03
				Patient 20	41.37
